# Scaffolding Mathematics Remediation for Academically At-Risk Students Following Developmental Education Reform in Florida

**DOI:** 10.1080/10668926.2017.1279089

**Published:** 2017-01-31

**Authors:** Rebecca L. Brower, Chenoa S. Woods, Tamara Bertrand Jones, Toby J. Park, Shouping Hu, David A. Tandberg, Amanda N. Nix, Sophia G. Rahming, Sandra K. Martindale

**Affiliations:** a Center for Postsecondary Success and the College of Education, Florida State University, Tallahassee, Florida, USA;; b State Higher Education Executive Officers Association (SHEEO), Tallahassee, Florida, USA

## Abstract

The purpose of this qualitative study is to understand how educational scaffolding may explain changing patterns of student success in mathematics in the era of developmental education (DE or remediation) reform in Florida College System (FCS) institutions. Specifically, we apply the concept of scaffolding to underprepared FCS students who are at risk of dropping out or failing gateway courses (the first credit-bearing college-level class in a course sequence) because they lack the academic skills necessary to succeed in college-level coursework, particularly in mathematics. We present data from focus groups conducted at 10 FCS institutions, suggesting that a reduction of scaffolding in math remediation occurred in the areas of course sequencing, instruction, and coordination with academic support services following state-level policy changes. In light of these findings, we offer a discussion of practical recommendations for college administrators related to academic advising, instructional strategies in DE courses, coordination between developmental and college-level instructors, student success courses, and the integration of DE instruction with academic support. We also suggest directions for continued research on the effects of policy changes in the FCS and DE reform efforts across the country.

President Obama’s proposal to provide free tuition for the first 2 years of community college was closely linked to his ambitious goal that the United States would again have the highest proportion of college graduates of any nation by 2020. This policy proposal acknowledges that in order to increase the proportion of college graduates in the United States, policy makers and institutional decision makers alike must find ways to increase persistence and graduation rates at community colleges, particularly for academically at-risk students and others who can benefit most from college degrees (The White House President Barack Obama, 2016).

With open admissions policies and diverse student populations, community colleges have traditionally been regarded as agents of social mobility, especially for at-risk students (Cohen, Brawer, & Kisker, 2003; Rosenbaum, Deil-Amen, & Person, 2007; Skidmore et al., 2014). The designation “at-risk” originates in the K-12 literature (Bulger & Watson, 2006) and is typically used to indicate students who “are poorly equipped to perform up to academic standards” (Quinnan, 1997, p.31). In this study, we define at-risk community college students as those who are at risk of dropping out or failing gateway courses (the first for-credit college-level class in a course sequence) because they lack the academic skills or crucial knowledge necessary to succeed in college-level coursework, particularly in mathematics.

Many at-risk students are recommended for developmental education (DE) or pre-baccalaureate classes taken in college that do not count for college credit (Cohen et al., 014). Nationally, 51.7% of community college students enter remediation, yet only 22.3% complete the remediation sequence, and even fewer (9.5%) graduate within 3 years (Complete College America, 2012, 2016). When exploring barriers to community college, there has been ample work regarding at-risk students’ likelihood of passing developmental and gateway math and English (see Bailey, Jeong, & Cho, 2010; Goldrick-Rab, 2010). However, there is a unique body of research around math in particular, mainly regarding students’ limited preparation and perceived level of course difficulty or math ability (Reid & Moore, 2008; Zientek, Yetkiner Ozel, Fong, & Griffin, 2013).

The purpose of our study is to understand how scaffolding may explain changing patterns of student success in the era of Florida’s new DE reform. Our research question guiding the study is: How did scaffolding of math remediation explain changing patterns of success for academically at-risk students following the DE reform in Florida? We first discuss the context for our study and then present our conceptual framework and review of literature. After highlighting our research question and explaining the method and data sources, we explore a reduction in scaffolding of math remediation for at-risk developmental students. Finally, we offer a discussion of practical recommendations for college administrators and directions for continued research on the effects of DE reform efforts in Florida and across the country.

## Study context: Developmental education reform in Florida

Florida Senate Bill 1720 (SB 1720) is part of a larger national developmental education reform movement, whose aim is to improve outcomes for academically underprepared students by increasing the quality of remediation and accelerating the DE course sequence. Introduced in 2013, SB 1720 was fully implemented in fall 2014 (Hu et al., 2015). The rationale behind SB 1720 was to help underprepared students reach and succeed in college-level courses more quickly, either through bypassing DE altogether or through instructional strategies designed to propel students more quickly to credit-bearing college courses. For students who entered into ninth grade at a public high school in Florida in the 2003–2004 academic year and subsequently graduated or who were active duty military, the legislation made optional the use of placement tests to assign students into DE and required these “exempt” students to be given the choice to bypass DE entirely (Hu et al., 2015).

Additionally, the legislation mandated that developmental courses be taught using new instructional strategies. Under SB 1720, colleges were required to design new DE curricula inclusive of the modularized, contextualized, compressed, and corequisite course options (or modalities). Modularized instruction, one of the most widely adopted new instructional strategies in developmental math (Hu et al., 2015), involves breaking course components into smaller instructional units (i.e., typically computerized modules) so that students can focus on the specific set of skills in which they are deficient. Contextualized instruction is related to “meta-majors” or “a collection of programs of study or academic discipline groupings that share common foundational skills” (Senate Bill 1720 [SB 1720], 2013, p.28), yet were infrequently implemented across the FCS institutions (Hu et al., 2015). Compressed courses were specifically designed to shorten the length of time in the developmental course. Corequisite instruction, according to SB 1720, is “instruction or tutoring that supplements credit instruction while a student is concurrently enrolled in a credit-bearing course” (p. 28). Corequisite instruction may take the form of concurrent enrollment in developmental and gateway courses, or simply additional tutoring for a gateway course. In either case, the supplemental assistance is designed to improve student performance in the gateway course. In addition to the new exempt status for specific student groups, eliminating the testing requirement for these students and implementing new curricular strategies, the legislation required advising for all students, which may include providing information about relevant developmental coursework.

A descriptive analysis of student-level data capturing exempt first-time-in-college students compared the first year of the implementation of SB 1720 to the previous year. Results indicated that developmental math course enrollments decreased in 2014, by more than 19 percentage points, and developmental math pass rates decreased, by nearly 2 percentage points (Hu et al., 2016). Conversely, enrollment in Intermediate Algebra, the traditional gateway math course increased, by 13.4 percentage points. However, gateway math pass rates subsequently decreased, by 12.0 percentage points for students enrolled in Intermediate Algebra. Despite this, cohort-based pass rates, or the overall pass rate for first-time-in-college students entering FCS institutions in 2014, rose following SB 1720. Due to the increase in enrollments, between 2013 and 2014 cohort pass rates in gateway math increased, by 4.0 percentage points. One explanation for the increase in cohort-based pass rates is the influx of students enrolling in the gateway math course, providing more students the opportunity to be successful.

## Conceptual framework

Our conceptual framework is grounded in the theory of scaffolding, which arose from Vygotsky’s (1978) sociocultural theory of learning, and may be particularly relevant to the educational needs of academically at-risk students. While Vygotsky did not coin the term “scaffolding,” the concept grew out of his related theory of the “zone of proximal development” (ZPD). Vygotsky (1978) defined the ZPD as “the distance between the actual developmental level as determined by independent problem solving and the level of potential development as determined through problem solving under adult guidance or in collaboration with more capable peers” (p. 86l). While Vygotsky originally applied his theory of ZPD to the sociocultural development of children, it was soon applied by other scholars to a wide variety of developmental tasks for children and adults in a range of educational and learning contexts (Chaiklin, 2003).

Vygotsky (1978) described the process of “mediation” in which either a person or an organized educational activity assists a child in performing a learning task. Akin to Vygotsky’s mediation, the concept of scaffolding assumes that a more competent person assists a less competent person with a learning task. Like the scaffolding on a building, the assistance expands the less competent person’s ZPD until the scaffolding can be gradually removed when that person has developed autonomy as a learner and has mastered the task without assistance (Chaiklin, 2003; Meyer & Turner, 2002).

Bemprechat (1992) has proposed that at-risk students, in particular, need educational scaffolding because they may be less likely to receive academic socialization from their home environments. While the importance of scaffolding math instruction for at-risk students is well established in the preschool and grade school literature (e.g., Clements & Sarama, 2008; Fuligni, Howes, Huang, Hong, & Lara-Cinisomo, 2012; Starkey, Klein, & Wakeley, 2004), Wass, Harland, and Mercer (2011) argued that teaching practices that scaffold instruction should be more widely applied to college settings. In our study, we use the concept of scaffolding to better understand the changes to math course pathways and instruction for at-risk developmental students in community college settings.

## Literature review

In order to keep pace with global competitors and increase the proportion of educated citizens in the United States, policies and practices aimed at improving educational outcomes must also focus on traditionally underrepresented students. Many academically at-risk college students have low income (Adelman, 1999; Bulger & Watson, 2006), have attended low-performing and/or low-resourced schools (Wimberly & Noeth, 2005), have limited access to crucial learning tools such as computers and high-speed Internet access at home (Pew Research Center, 2013), and face extensive home and family obligations such as child rearing and full-time or part-time work (Engle & Tinto, 2008). Many of these factors are associated with decreased odds of completing a college degree, particularly a bachelor’s degree (Choy, Horn, Nuñez, & Chen, 2000; Engle & Tinto, 2008).

### Barriers to success for academically at-risk students

Many at-risk students face barriers over which they have little or no control. Research indicates that under-resourced or low-performing high schools, lack of consistent computer and Internet access, and limited access to other resources such as transportation or affordable textbooks are just some of the barriers these students face. For example, many underprepared students attend failing, low-performing, or under-resourced high schools (Adelman, 2006; Glazerman & Max, 2014) where lower-performing students are offered limited or no access to a high school counselor (Kimura-Walsh, Yamamura, Griffin, & Allen, 2008; Woods & Domina, 2014), to Advanced Placement (AP) courses (Solorzano & Ornelas, 2004), or to high-quality or more experienced teachers (Glazerman & Max, 2014; Socias, Chamber, Esra, & Shambaugh, 2007). These factors, in combination with a solid academic preparatory sequence and math performance in particular, can influence students’ success in college (Adelman, 2006; Conley, 2008). Research indicates that students with access to high school counselors are better situated to be admitted to and succeed in college (McDonough, 1997; Woods & Domina, 2014). Further, DE outcomes specifically are related to students’ high school academic program; students who complete an academic core curriculum in high school were less likely to need remediation (Ohio Board of Regents, 2002). Similarly, students who enrolled in developmental math courses had enrolled in fewer high school math courses, earned lower high school grades in math, and had lower math-specific and overall scores on the ACT (Bettinger & Long, 2005).

For at-risk students who also have low income, access to a computer and/or Internet access at home may be limited. Across the country, 78.1% of Americans had high-speed iInternet access at home (File & Ryan, 2014), but only 52% of those in households earning less than $30,000 per year had broadband Internet access at home in 2013 (Pew Research Center, 2013). Computer use has also been linked to better educational outcomes for community college students and may be particularly helpful to those who live further from campus (Fairlie & London, 2012). These issues are particularly relevant when many classes are offered either completely or partially online. Additionally, many scheduling and registration activities occur online, and having access to a personal computer and at-home IInternet access can help students register on time and avoid late registration fees and courses that are full and closed to additional students.

At-risk students may benefit from individualized support services and guidance to overcome these challenges, particularly in the face of new enrollment and registration policies. As part of a revolutionary wraparound program taking place among several City University of New York (CUNY) institutions, the Accelerated Study in Associate Programs (ASAP) offered many benefits such as advising, career services, and tutoring; linked courses and several-semester seminars; tuition waivers to fill gaps between financial aid and required fees; and free public transportation and use of textbooks to combat low-income students’ barriers to success (Scrivener et al., 2015). Although the program was initially costly, it was well implemented, and graduation rates nearly doubled after 3 years. In fact, the program actually reduced the per-graduate cost for ASAP participants, as compared to nonparticipants. Such a widespread program may not seem feasible to many college administrators, but there are aspects of the program that can be easily scaled. Aside from comprehensive, large-scale programs such as ASAP, teaching pedagogy, including scaffolding, can also assist students’ learning.

### Educational scaffolding

Academically at-risk students may need additional programming or scaffolding to aid their educational success. Scaffolding can support a variety of learning objectives including absorbing course content and concepts, increasing self-awareness, providing motivational support, understanding how to use learning and teaching tools such as computerized learning platforms, and learning techniques to adapt to different instructional contexts (Azevedo & Hadwin, 2005; Meyer & Turner, 2002). Below we discuss scaffolding in course sequencing, course instruction, and academic support services.

#### Scaffolding in course sequencing

There is some evidence that scaffolding students’ course sequences and the course registration process is beneficial to students. Rosenbaum et al. (2007) suggested that colleges offer a small set of options, combined with closely linked support services. Similarly, Scott-Clayton (2011) argued that providing students with too many course enrollment options may result in poor choices. The author recommended that schools not restrict choices, but provide a “‘prixe-fixe’ menu, offering a limited selection of prepackaged college pathways,” or “‘smart defaults’” which offer students a launching pad for the registration process (Scott-Clayton, 2011, p. 26). It is also possible for students to create custom degree maps in which colleges offer structured degree programs that students can tailor to their educational goals with the help of an advisor (Bailey, Jaggars, & Jenkins, 2015). Advisors need to create intentional course scheduling opportunities that provide clear course pathways matched to students’ motivation and goals (Muraskin & Lee, 2004; Schreiner & Anderson, 2005). Indeed, Bahr (2008) found that advising is positively related to student success and may be especially effective for low-performing students who may be navigating multiple levels of developmental math.

#### Scaffolding in course instruction

Scaffolding has been used in math education and in college math course settings in particular. Instructional scaffolding is defined as the “provision of guidance and support which is increased or withdrawn in response to the developing competence of the learner” (Mercer, 1995, p. 75). Scaffolding can be implemented in different ways, including through teacher practices and pedagogy or through supplemental supports and instruction outside of a traditional class setting.

Mathematical learning involves acquiring both conceptual knowledge (or understanding relationships among pieces of information) and procedural knowledge (or understanding symbols of representation and rules for completing math tasks) (Hiebert, 2013). In the context of math instruction and students’ understanding of mathematical proofs, Blanton Stylianou, and David (2003) stated that “students’ capacity to engage in these types of discussions [about mathematical proofs] …can be scaffolded” through effective teaching practices (p. 2–119). Meyer and Turner (2002) explained that the specific process of scaffolding mathematical learning involves breaking down problems into discrete parts while providing students with partial solutions and hints as necessary. This assistance is gradually reduced as students attempt new problems with increasing levels of autonomy and encouraged to articulate their solutions while providing rationales for their approach. A non-scaffolded approach to math instruction would not foster greater student autonomy. Instead, it would involve asking only for solutions and requiring rote calculation without any check on whether students understand the methods used to solve the problem. This approach would offer few opportunities for students to check their understanding of the overriding mathematical principals behind problems.

Scaffolding can also occur in computer-based or online formats. Quintana, Zhang, and Krajcik (2005) proposed a framework of scaffolding which can assist students in their online inquiry tasks (e.g., projects where students use the Internet to address their questions). Their framework is specifically designed for novice learners, many of whom may be students enrolled in DE courses. Further, given the increased presence of online learning under Florida’s DE reform, scaffolding for these types of projects is particularly relevant. As summarized in their review of the importance of scaffolding, Azevedo and Hadwin (2005) noted that learning about complex topics in computer-based learning environments without scaffolding may lead to students’ inability to understand the topic. Therefore, the authors argued that research has begun to stress the importance of embedding various types of scaffolding into these learning environments. Even within computer-based learning environments, scaffolding and hints on math problems can originate with the instructor, the computer program itself, or classmates (Azevedo & Hadwin, 2005).

#### Scaffolding in academic support services

Student success courses serve as an introduction or orientation to college. The benefits of participating in these courses include the opportunity to gain important information about college resources and courses, develop study skills, form relationships, and gain knowledge of college resources (O’Gara, Karp, & Hughes, 2009). In addition, these courses encourage student use of college resources and facilitate stronger advising relationships (O’Gara et al., 2009). These orientation classes act as a way to scaffold new students’ college awareness, readiness, and college knowledge, or the “softer skills” which include study skills, collaboration techniques, and an understanding of college culture that contribute to college success (see Conley, 2008). These “soft skills” are especially important in educational scaffolding due to the socio-emotional and motivational benefits they provide students (Meyer & Turner, 2002).

## Methods

The current study is part of a larger mixed-methods policy analysis of SB 1720 (Hu et al., 2015). In this study, we employed qualitative data collection and analysis methods. Our qualitative sampling strategy was a purposive, maximum variation sample involving “purposely picking a wide range of cases to get variation on dimensions of interest” (Patton, 2015, p. 243). The 10 institutions in our sample represented every region in the state of Florida. The community colleges also differed in terms of enrollment sizes; rural, suburban, and urban locations; and performance on student outcome variables as measured by our quantitative dataset. Prior to data collection, our research was approved by our institutional review board and those of the institutions we visited where required.

### Data collection

We completed two-day site visits to the 10 FCS institutions in fall 2014 and early spring 2015. Data sources included field observations, institutional documents, and transcripts from focus groups with relevant stakeholders at the institutions. Institutional documents related to implementation such as PowerPoint presentations, advising flowcharts for course sequences, and new student orientation materials were collected during site visits. Researchers also generated field notes for each site visit, identifying salient, interesting, or illuminating observations.

Focus group participants were diverse in their roles at the community colleges we visited. We conducted 87 semi-structured focus groups lasting between 20 and 111 minutes with 5–10 individuals. In total, we spoke with 78 administrators, 140 faculty members, 71 academic advisors, 25 support staff members, and 204 students, resulting in data from 518 focus group participants. On average, we interviewed 52 participants of all types per institution, with a range of 39 participants at one small institution to 74 participants at a large institution.

As part of a larger mixed-methods study of SB 1720, each of the five interview protocols began with a “grand tour question” (Spradley, 2016) intended to identify broad changes in institutional practice after the passage of SB 1720. The administrative protocol, for instance, began with the following question, “Overall, how would you describe your college’s approach to redesigning your developmental education program?” Likewise, the faculty, advisor, and support staff protocols began with grand tour questions eliciting an overview of curricular changes, changes to the advising process, and changes to academic support functions. The student protocol focused on students’ perceptions of institutional changes in several areas including curriculum, advising, and support services. From these opening questions, follow-up questions were then asked for participants to elaborate on their perspectives about implementation of the legislation, which included specific questions about challenges and barriers participants had experienced with the institutional changes as well as the effectiveness of advising, the four developmental instructional strategies in math, the gateway math curriculum, and academic support services. Each focus group concluded with the following question, “Is there anything we didn’t ask about developmental education reform at your institution that would be important to know?”

### Data analysis

Our qualitative data analysis process included five phases, involving a combination of both a priori and emergent coding. Throughout the data analysis process, the coding team of five analysts met weekly to share findings and discuss emergent themes. In the first phase of analysis, we read through the field notes, institutional documents, and focus group data to synopsize the chronology of policy implementation processes at each institution. A chronology of the implementation process was then written for each institution. In the second phase of analysis, we identified key themes which arose from implementation at each of the 10 FCS institutions. Based on the themes in our synopses of the implementation process, an a priori coding framework was developed that included 157 codes. The codes included broad parent codes like “student populations” and “curricula” and more detailed child and grandchild codes like “low-income students,” “developmental math,” “gateway math,” and “instructional quality” (Bazeley & Jackson, 2013).

In the third phase, we imported our focus group interview data and coding framework into qualitative data analysis software, NVivo 10, for coding. Coding first involved a subset of eight data files for a reliability-building process. The a priori framework was then used to achieve inter-coder reliability.

In the fourth phase of analysis, we used pattern coding of the focus group transcripts to analyze the remaining data. Through this process, we identified additional emergent themes not captured under existing codes, resulting in 54 additional codes. (Corbin & Strauss, 2014; Miles, Huberman, & Saldaña, 2014). The qualitative research team wrote analytic memos (Corbin & Strauss, 2014) throughout the coding and analysis process.

In the final interpretive phase of analysis, scaffolding for math remediation emerged as a prominent theme, and we confirmed that this theme appeared in our data across all 10 institutions. We further delineated scaffolding into three subthemes: (1) scaffolding of the math course sequence, (2) scaffolding of the math curriculum, and (3) scaffolding of academic support for math instruction.

Trustworthiness was established through triangulation, member-checking, and peer debriefing. Five analysts coded the data in order to achieve analyst triangulation and three data sources were used (field notes, institutional documents, and focus groups) for data source triangulation. Additionally, four researchers engaged in peer debriefing by acting as “devil’s advocates” in questioning the group’s interpretations (Patton, 2015). We conducted member checking by soliciting feedback on our research products from administrators at each of the 10 institutions. After incorporating the feedback of administrators, we distributed our revised research products to all focus group participants who had provided an email address.

As with every qualitative study, the setting for our study was unique. The higher education landscape, the organization of the FCS, and the policy environment of DE reform provided an ideal context to study changes in remediation for at-risk students. However, within this unique context, our study has several limitations. The institutions in our sample were those that accepted our invitation to conduct research on their campus, and focus group participants were those identified by institutions, both of which may have influenced our findings. Nonetheless, we maintain that the number of institutions, the types of participant groups, and the total number of participants (518) ensured that our study incorporated a breadth and depth of perspectives from across the FCS. In addition, we acknowledge that future shifts in institutional practice toward greater scaffolding of gateway math classes could result in improved student outcomes in these courses. Despite this, we contend that understanding scaffolding of math remediation for at-risk students has equal relevance for institutions that are moving toward reducing their DE course offerings and those with robust DE programs.

## Scaffolding math remediation for academically at-risk students

We present here the perspectives of campus personnel and students on the decreased scaffolding of math remediation following DE reform in Florida. Our qualitative data suggest that following DE reform, scaffolding for math remediation decreased for at-risk students in three ways: (1) math course pathways became less scaffolded, (2) students who opted to bypass developmental math courses received less scaffolding from instructors in gateway math courses, and (3) students who opted to bypass developmental math courses received less scaffolding from academic support services that were less closely integrated with gateway math courses.^1^ We first consider specific challenges of at-risk DE students and then examine these three ways in which scaffolding was reduced.

### Barriers to success for at-risk DE students

Campus personnel in our focus groups identified a number of challenges that hamper the academic success of at-risk and low-income developmental students. Challenges faced by at-risk developmental students who participated in our study included significant financial limitations, family commitments, a lack of reliable transportation, and limited access to basic personal and academic needs such as glasses and Internet access at home. Such challenges meant that these students tended to need more educational scaffolding than their peers. Faculty members in one of our focus groups discuss many of these barriers:

Faculty member 1: Well, our campus is about 80% that are below the poverty line or close. So that’s huge because of all the outside factors. Most of them work. They can’t—most of them are obviously on financial aid; they get full financial aid, but they don’t have a car, they have to take the bus, you know, they—a lot of ‘em, they come to school hungry, and they’ve got kids….
Faculty member 2: Right now, I’ve got one that he can’t see any—like, from me to you, he can’t see the board and he can’t afford to get glasses, you know?…
Faculty member 1: Yeah, and even basic supplies for school. I collect used notebooks and stuff just to give away to my students and dividers. I go buy calculators for them because they can’t—they don’t have the money for it, you know? They jump from paycheck to paycheck, so I think low SES is huge.
Faculty member 2: The issue with Internet access and not having a computer, you know, depending on the bus, transportation issues, those are…

Another faculty member stated that the students “don’t have money sense” and “don’t know how to prioritize” their finances. Students will sell their textbooks back in the middle of the semester to pay their bills and use the money “like floating a loan” only to rebuy the textbook the next month to catch up on their coursework. These examples explain how students’ financial situations can interfere with their learning and academic progress, often because of limited access to resources.

### Scaffolding the math course sequence

Exempt developmental math students were faced with two new choices when selecting courses: (1) bypassing developmental math altogether or (2) choosing from new instructional strategies in the developmental math courses that remained. Institutions were required to offer a minimum of two of the new DE instructional strategies, yet some institutions adopted all four. Exempt students were faced with a number of enrollment options, many of which were confusing to college personnel and students. For instance, a faculty member remarked, “We knew we had to have at least 2 of the modalities and… I think we ended up doing all of them to some degree.” A faculty member at another institution commented that, “There are 247 different prep [developmental] classes being given around the state.” As an example of the complexity of the new course pathways, faculty members described students’ math options at their institution:

Faculty member 1: The first developmental class is the MAT 0018, which was called Pre-Algebra. Now it’s Developmental Math 1. And then the next one is MAT 0028, it used to be called Beginning Algebra and now it’s Developmental Math 2. And now I guess we have kind of two different tracks now. The Gateway Algebra MAT 1033 is considered a college prerequisite credit but not math credit. And then you get up into the first college-level Algebra class….
Faculty member 2: There’s an STA 1021. So after the 18. 18 is prerequisite for the STA soon to be 1001, which is a statistical reasoning course and after the STA, again, now it’s 1021 it will be 1001 in the fall, then they can go to the regular college-level statistics, which is the STA 1023 and those liberal arts classes the MGF 1106 and 1107….

Perhaps not surprisingly, campus personnel and students alike reported significant confusion about the proliferation of course options in the new math sequences. Faculty and advisors consistently argued that developmental math students needed the scaffolding of clear course pathways and fewer course options rather than the expansion of course options that occurred under SB 1720. Whereas interviews with advisors clearly revealed their efforts to guide students into the appropriate math course, many students rejected their advice. An advisor described her frustration when speaking with students about the importance of building a solid foundation in their understanding of basic skills. However, the advisor also noted that, “If you tell them you don’t need to take these classes, the automatic response is, ‘I don’t want to.’” Indeed, as evidenced by the decrease in pass rates between the 2013 and 2014 cohorts, many exempt students chose to enroll in MAT 1033 despite being unable to successfully complete the course (Hu et al., 2016).

In addition, campus personnel believed that students opted out of DE math courses because they were less able to assess their own level of academic preparation in math and wanted to save money. An advisor commented, “Generally the student consumer mentality is that I need to save myself time and money. Or I don’t need that because that’s not a part of my degree program, and I’ve had that.” Confirming this perspective, a student explained her decision to enroll directly in Intermediate Algebra rather than take developmental math courses:

I just think that they’re all—I think it’s not necessary, in my opinion, because the difference between MAT 0028 and 0029 and 1033 is, it’s all the same thing. If you have the right professor for 1033 that you have for 0028 or 0029, it’s the same thing. It’s the same exact information. It’s just set at a little bit slower pace. So if you were to take a full semester of let’s say 1033, you can get the same amount of information that you would get from two semesters, one being 0028 and one being 0029. So I think that they’re not necessary.

What is unclear, however, is how successful this student and others similar to her are in their gateway courses. For many at-risk students, opting to skip developmental math courses and enroll directly in Intermediate Algebra may not have served them well because these students tended to perform better academically in developmental math courses (Hu et al., 2016).

### Scaffolding of the math curriculum

One of the possible explanations for at-risk students’ stronger academic performance in developmental math courses was the scaffolding provided by the developmental math courses. At one institution, for instance, an administrator described how various instructional strategies offered differing levels of scaffolding for students of varying academic preparation:

Students come in really underprepared, students come in and they are right where they should be for that, or they come almost ready for the next. So each of our levels basically has three courses, and they are designed to meet each of those tiers. So, the least prepared get the full blown contextualized course. The middle ones get opportunity to accelerate by taking a compressed combined course. And, then the ones on the edge get the modules so they only do what they need to do, at their own pace, but facilitated by an instructor.

The administrator emphasized that offering various levels of courses was intended to best help students succeed efficiently.

Developmental math courses were scaffolded both in terms of the design of the curriculum and in the ways in which developmental math instructors presented the material. Two instructional modalities, in particular, modularized and corequisite instruction, were carefully scaffolded. Modularized instruction offered students the ability to work at their own pace (typically online) and accelerate their learning while corequisite instruction allowed students to gradually prepare for college-level math by combining the developmental and gateway math course. A faculty member described how he used the modularized curriculum to assess students’ difficulties with specific math problems:

It allows for one-on-one help…. So I can see how many—how long they’ve been on [the online program], see how many topics they’ve attempted, and what they’ve actually gotten right. So I had one kid that had been on for 16 minutes, one topic attempted, zero right. I went into his account, saw how many times he had hit explain or saw how many times he had gotten it wrong… and we did one-on-one help. I corrected his mistakes. Right at my desk, I said, ‘okay, click on the next problem and let’s do it.’ He did it by himself, I sent him back to his desk, and then a little bit later I checked that he mastered that topic.

We also do some group discussions in class. So I have three students in that class who have attempted the same topic, but haven’t mastered it, the system will tell me. I bring them up, and I give them a quick ten-minute tutoring on that system, on that topic, send them back, and let them do the problems themselves.

Students also shared their perspectives on the developmental math curriculum. One student described her initial concerns about enrolling in a modularized developmental math course:

I’m taking 0057…They said that it’s two maths combined. Before the course started, I went to the bookstore to get my book and they gave me a card called ALEKS (Assessment and Learning in Knowledge Spaces) and I’m like ‘now where is the book?’, and it was like ‘oh, it’s all online.’ I was gonna go crazy because I don’t take online classes because I just can’t sit online and do what I’m supposed to do…. So I started ALEKS and I actually like ALEKS. It explains step by step and it gives you multiple questions that you can continue to practice, and it moves you forward at your own pace. So, so far, math is working out well for me. And like you said, I was rusty as well, and then when ALEKS explained it, I’m like, ‘oh, I remember this, yes, this is how you do it.’

After her initial apprehension about the online delivery method, the student described how the online scaffolding helped her succeed in the course.

The corequisite instructional strategy was also scaffolded to encourage students to become increasingly autonomous in studying the course material. An instructor described her corequisite math course:

I have to start breaking away, like holding the hand. Because once 8 weeks passes, now I’m gonna consider you in our college class…. So I mean, I have to give them a slow transition. And as the 8 weeks progresses, the lectures get longer and longer and longer. And by the end, that’s it. It’s full lectures the entire class, and they have to be on their own because that’s the only way they’re gonna be successful is when they go out in the business world. No one is gonna be holding their hand. I’m like, ‘Look, we’re gonna start here nicely, but by the end, you’re gonna be doing everything on your own.’

The instructor described how she gradually decreased scaffolding over the course of the semester until students were prepared to work independently. The student perspective reflected a similar process of increasing difficulty as the semester progressed. A student described the scaffolding of her DE math course content:

I’m completely satisfied with the classes that I chose and the classes that I placed in [DE math], because it’s at my level. It’s something that I comprehend and understand…because gradually as time goes on things are gonna get a little bit more difficult along the way. Things are gonna get a little bit more complex, but that’s how it’s supposed to be.

Data from both campus personnel and students suggested that DE coursework was designed to facilitate a reduction in scaffolding and an increase in independent learning.

In addition to the scaffolding built into the developmental math curriculum, our focus group participants indicated that developmental instructors often adopted a different teaching philosophy than gateway instructors. An administrator explained the different approaches in developmental and gateway instruction:

Learning how to be a student—that was one of the things that I really saw the students learned when they were in the developmental courses. They understood how to persist. There was a nurturing environment too to help students to grow in that direction. Whereas when you go more straight into the credit course, there is a different expectation that you have a little more self-motivation, and you understand how to manage time and those things…. So when you take your first courses, and you’re not really quite ready for college, or really college credit, you have to learn how to swim in the deep end right away. And so there is nothing there to help them segue from one to the next in terms of—and I’m thinking of the kinds of structured activities [in developmental courses] and so forth.

Not only were developmental math instructors more helpful with struggling students, but also incorporated critical study skills and “college knowledge” to increase students’ readiness for college-level coursework.

In contrast, some focus group participants worried that instructors in gateway math courses were less likely to provide crucial scaffolding to academically underprepared students. An advisor described the philosophy of many gateway math instructors she had encountered:

They [gateway instructors] said ‘These students are not going to be prepared for our classes. What do we do with them? We are not going to go back. We are going to teach like we always teach. We are not going to do anything special for these students.’ We [the advisors] said, ‘We understand that, and we can pass that on to our students that there is nothing special that will be happening. [The instructors] are going to expect a certain base of knowledge coming into the class and you might not have that.’

Advisors had the difficult job of communicating the rigor of gateway courses to students who were sometimes unreceptive to this message. Therefore, advisors provided students with resources such as opportunities to receive tutoring, particularly since it was clear that gateway course instructors were not planning to review concepts that they believed should have been previously mastered. Similarly, a faculty member expressed his concern about the “sink or swim attitude” in college-level courses and how that might negatively impact success for at-risk student populations:

I just worry about that sink and swim attitude, you know. I wonder if we even know—and, I guess we will know when we see the fails and we see the withdrawals, but are we reaching people with learning differences? Are we reaching people with learning disabilities? Are we reaching people from socioeconomic backgrounds where they have to start from ground zero, not for any reason of their own?

Echoing this sentiment, an exempt student who had opted out of DE described a professor’s sink or swim attitude in her gateway course:

“And you’re supposed to know this already.”… It really made me pissed off with that professor [when she said that], so like I left the class that day. Okay, well, some people learn slower than others, you know.

The student was offended and frustrated when her instructor informed her that they were covering eighth grade material. Describing the gateway instructors, another student explained, “I just want the teachers to be patient.” Further, many campus personnel expressed concern for students such as these, especially those with extenuating life circumstances and the greatest need for remediation. In addition to scaffolding of the math course sequence and instruction, scaffolding of academic support was also essential to the success of at-risk math students.

### Scaffolding of academic support for math

Another important aspect of scaffolding math remediation is offering a comprehensive, coordinated approach to academic support services. One example of such scaffolding is the early alert system that many institutions used to help faculty and advisors identify at-risk students. A faculty member described how early alert, a system designed to “raise a red flag,” functioned at his institution:

When I submit an early alert in a day or two somebody from advising will write to me and will let me know, we have contacted the student and these are their results. Then I have had students, as a result of that, email me or send me a text: ‘My adviser contacted me about your class. What can I do to improve?’ So there have been those success stories that I see the system working and going full circle from my raising the red flag, to advising contacting me, to the same student then contacting me saying they were contacted. I would say that’s a great system. It’s definitely working for my classes.

Whereas the early alert system employs communication first between the instructor and the advisor, then with the student, embedded (or in-class) tutors work directly with students first. With respect to academic support staff, a faculty member described how embedded tutors coordinated with faculty to scaffold instruction in developmental courses:

An embedded tutor…was just someone in the classroom that could answer student questions while the professor was …having small group discussions… So the idea of bringing a person to the class to talk to them from the lab gives them that beacon of hope when they open the door and they know that that person’s gonna be from this time to this time…they can see that person—‘that person that came to my class and talked to me. You know, I can relate to that person and go to that person….’ It’s learning how the students are being taught inside the classroom and then replicating that same style in the laboratory.

The embedded tutors who also work in the learning labs understand how and what the instructor is teaching in class and therefore can provide similar supplementary assistance in the lab after class ends. However, our data suggested that higher levels of coordination between faculty and support staff existed for developmental courses than for gateway courses. An administrator explained:

All of the developmental ed courses…required students to go to the learning commons, which is our tutoring center. And I think—and I know their numbers in their participation over there have gone down significantly [since students were able to bypass developmental math]. But I think when students were required to go there as part of their developmental courses, they got used to going there. They saw the value of it. They got the help they needed.

The same level of coordination may not have existed between gateway instructors and support staff. Further, students were more likely to use the support services when they were required as part of the course.

## Discussion

Our study differs from previous studies of at-risk community college students in several ways. First, we apply the concept of scaffolding, which is more prevalent in the K-12 literature (Wass et al., 2011), to a new setting: math instruction for academically at-risk students in community colleges. In addition, while many previous studies of at-risk students have considered the effects of specific programs and practices on student success, our study considers scaffolding more holistically as a process that incorporates not just curriculum but several aspects of the student experience including advising, classroom practices, and academic support. Lastly, we consider scaffolding of mathematics remediation in the context of a specific policy change (e.g., developmental education reform), a movement which is gaining traction in community colleges nationally (redacted).

While the intention of SB 1720 was to accelerate the DE sequence and improve educational outcomes for all developmental students (redacted), our study suggests that at-risk students may be poorly served by policy directives allowing them to bypass developmental courses altogether, at least in math. Our findings confirm previous research (Rosenbaum et al., 2007; Scott-Clayton, 2011), suggesting that greater student choice in math course pathways is not necessarily beneficial for some students.

At-risk students in need of math remediation may have benefited from specific features of the redesigned developmental math instruction in FCS institutions. Specifically, our data suggest that the developmental math curriculum was more scaffolded both in terms of instructional strategies and in the teaching philosophies of developmental instructors. In addition, we found greater integration of academic support services in developmental math courses than in gateway math courses. Our findings suggest a number of institutional practices which can benefit at-risk community college students.

### Recommendations for institutional practice

Our focus group data suggest that math instruction for developmental students can be scaffolded in terms of the course sequence, the instructional strategies utilized, and coordination with academic support services. Indeed, institutions with high graduation rates tended to offer “intentional academic planning” which ensures that students enroll in a planned sequence of courses (Muraskin & Lee, 2004, p. 3). Advising should also be designed around “informed course selection” that complements the student’s strengths, academic preparation, interests, and future plans (Schreiner & Anderson, 2005). Further, advisors can be embedded within certain career pathways to more closely link students’ skills, interests, and courses with specific career fields (Bailey, Jenkins, Belfield, & Kopko, 2016). Previous research indicates that academic advising may be more effective for lower-prepared students, compared to their higher-performing peers (Bahr, 2008).

Because mathematical learning is sequential, particularly in procedural learning (Hiebert, 2013), knowledge transfer from one course to another is essential. Greater coordination among instructors promotes knowledge transfer (Benander & Lightner, 2005). Knowledge transfer in math can improve when there is greater coordination between DE instructors and gateway instructors. Coordination can be accomplished by scheduling the same instructors to teach developmental and gateway math courses. This arrangement also ensures that gateway instructors have experience working with developmental students and understand how to scaffold their college readiness needs. When scheduling the same instructor is not possible, it can be beneficial for developmental and gateway instructors to collaborate and confirm that developmental math instructors are preparing students for the skills they will need in gateway courses and that gateway instructors understand the unique needs of at-risk students when they enroll in college-level coursework. In addition, coordination between developmental and gateway math instructors is more integrated when DE faculty are members of the math department rather than a standalone DE department.

Modularized and corequisite developmental math instruction may be particularly well suited to scaffolded learning tasks for at-risk students. Modularized math courses (typically offered in an online format) allow students to work at their own pace and focus on practicing only the skills for which they need extra assistance. Self-paced modules can allow for more student control over their learning and the ability to return to difficult units, with the scaffolded help of the instructor, until mastery is attained (Bickerstaff, Fay, & Trimble, 2016). Corequisite math courses, which gradually increase in difficulty and decrease in instructor scaffolding as students progress to college-level coursework, may be a good option for students who prefer face-to-face instruction (Hu et al., 2015). This instructional modality provides “just-in-time” support with additional class or lab sessions (Complete College America, 2016).

We also recommend that colleges continue to offer high-quality student success courses or first-year seminars. Previously, many FCS institutions had student life skills (SLS) courses required for DE students, but since implementing SB 1720, some colleges have eliminated the requirement. Although Permzadian and Credé (2015) found only small effect sizes on increasing first-year GPAs and first-year retention rates for similar courses, they provided recommendations for improving the courses’ success. Courses should include all students, not merely those with limited academic preparedness; have faculty, as compared to undergraduate or graduate students, instruct the courses; and focus on orienting the students to college by providing the relevant college knowledge that Conley (2008) recommends.

Another type of scaffolding for math is the close integration of math instruction with academic support. Academic support services should be offered in multiple methods. In-class (or embedded) tutors, learning labs and centers, online tutoring with modules and live support, and peer mentoring programs offer the most access for at-risk students (Bailey et al., 2015; Hu et al., 2015; Perin, 2004). Coordination between academic support staff and instructors can be facilitated by the use of embedded tutors who are known to students because they attend class and discuss the math curriculum with the instructor; by having instructors require hours in the lab and holding their office hours in the lab; and by scheduling math courses so that they are physically located in close proximity to learning labs. Similar to the recommendation of offering courses at a variety of convenient times and at multiple campuses, students who live near auxiliary campuses should be afforded the same level of academic support services offered at the main campus.

### Future research directions

Institutional policies and practices in Florida community colleges are not static. Preliminary qualitative data findings from our continued evaluation in 2015–2016 suggest that administrative priorities at many FCS institutions have shifted from redesign of the DE sequence to focus on the improvement of gateway courses. Therefore, further qualitative analyses are needed to determine whether this shift in institutional focus in tandem with efforts to incorporate more scaffolding strategies in gateway math will result in improved student outcomes. Our future work will also compare the effectiveness of different instructional strategies (i.e., compressed, contextualized, modular, and corequisite instruction) that have been adopted in developmental math courses in Florida.

Furthermore, a review of studies that reduce or eliminate DE in other states is necessary to determine whether this national trend will result in improved student outcomes for all academically at-risk students. Longitudinal research on DE reform in Florida and other states is needed to determine the effects on graduation and transfer rates overall and for specific underrepresented student populations. Lastly, studies in other states that identify promising practices for at-risk developmental students will further inform our future research plans and practical recommendations for community colleges in Florida.

## Conclusion

As a nation, we cannot dramatically increase educational attainment rates for our knowledge-based economy without first improving student outcomes for at-risk student populations at the community colleges that enroll a majority of postsecondary students in the United States (The White House President Barack Obama, 2016). Furthermore, because developmental math is often a stumbling block to success for these students (Attewell, Lavin, Domina, & Levey, 2006; Bailey et al., 2010), we must work harder to meet the need for math remediation through comprehensive efforts to scaffold the curriculum. In this study, we have presented qualitative findings on scaffolding math remediation in the FCS after the implementation of SB 1720. Our findings lead us to recommend that institutional decision makers and campus personnel who work directly with at-risk students improve the scaffolding of math remediation by focusing on clear course sequencing, a structured math curriculum, and a more seamless integration of support services with classroom instruction, particularly within gateway math courses.

## Funding

The research reported here was supported by the Institute of Education Sciences, U.S. Department of Education, through Grant R305A160166 to Florida State University, and in part by a grant from the Bill & Melinda Gates Foundation. The opinions expressed are those of the authors and do not represent views of the Institute or the U.S. Department of Education or the Gates Foundation.

## Footnotes

1 Note that in our data, course prefixes beginning with MAT 0 indicate pre-baccalaureate developmental math courses, whereas MAT 1033 indicates college-level Intermediate Algebra (the gateway math course).
